# Early musculoskeletal classroom education confers little advantage to medical student knowledge and competency in the absence of clinical experiences: a retrospective comparison study

**DOI:** 10.1186/s12909-018-1157-7

**Published:** 2018-03-27

**Authors:** Derek Khorsand, Ansab Khwaja, Gregory A. Schmale

**Affiliations:** 1 Department of Interventional and Diagnostic Radiology, University of Washington Medical Center, Health Sciences Building, RR210, 1959 NE Pacific Street, Box 357115, Seattle, WA 98195-7115 USA; 2 Department of Orthopaedic Surgery, University of Arizona, 1609 N. Warren Ave, Suite 110, Tucson, AZ 85719 USA; 3 Department of Orthopaedics and Sports Medicine, University of Washington School of Medicine, Seattle Children’s Hospital, PO Box 5371, 4800 Sand Point Way NE, Seattle, WA 98145-5005 USA

**Keywords:** Musculoskeletal medicine, Musculoskeletal education, Medical education

## Abstract

**Background:**

Deficiencies in medical student knowledge in musculoskeletal medicine have been well documented. To address these deficiencies, numerous curricular changes at our institution were instituted. The objective of this study was to determine whether medical students in their preclinical years benefit from early exposure to musculoskeletal medicine when compared to students exposed to musculoskeletal medicine just prior to completion of their preclinical curriculum.

**Methods:**

United States Medical Licensing Examination (USMLE) Step 1 and 2 scores were compared for periods of time before and after institution of the new curriculum. Scores on the previously validated 24-question short answer survey from Freedman and Bernstein were also compared over these same periods of time between these two groups and to established standards for competency, using a student’s two-tailed unpaired t-test for significance. Entering Medical College Admission Test (MCAT) scores were used to compare baseline preparation of students.

**Results:**

Overall USMLE scores as well as scores on the USMLE subtest on Musculoskeletal, Skin and Connective Tissue Disease showed no improvement when scores were compared between the two groups of students. There was a statistically significant lower performance on the Freedman and Bernstein knowledge assessment exam for students in the new pre-clinical curriculum as compared to those introduced under the old model, considering both musculoskeletal knowledge (*p* < 0.001) and proficiency (*p* < 0.01), though the response rate on the recent survey was low (112/986 or 11%). Spine remained the least understood sub-topic, while a dedicated course in rheumatology likely contributed to increased student knowledge in that area. Additional exposure to musculoskeletal topics during the clinical years increased student knowledge. There was no difference between groups when comparing entering MCAT scores.

**Conclusions:**

Classroom curricular changes, including moving the introductory musculoskeletal course to the first year, intended to optimize musculoskeletal medicine education in the pre-clinical years of medical school did not appear to improve student musculoskeletal knowledge at any year of training. Further efforts to improve the education of medical students in musculoskeletal medicine should be directed towards providing more clinical experiences with patients having musculoskeletal concerns. This was a retrospective comparative study, level III evidence.

## Background

Musculoskeletal problems number among the most frequent seen by primary care providers [1–4], yet US medical school instruction in musculoskeletal medicine has been found to be absent [4–6] or inadequate [7–13] by many. Low confidence in treating musculoskeletal problems and performing basic treatments for these complaints was seen in surveys of many primary care physicians [14–17]. Reported changes to medical school pre-clinical curricula in musculoskeletal medicine to address these deficiencies have included introducing basic introductory courses where none previously existed [18, 19], designing courses that mixed small groups with large-group lecture instruction, including instruction in physical exam [13, 18, 20], using interactive seminars where small group instructors were few in number in place of or to supplement didactic lecture sessions [21–23], and providing instruction in clinical history-taking and physical examination to a degree allowing for student evaluation using Objective Structured Clinical Examinations to encourage and evaluate student progress [24–26].

Highly successful musculoskeletal medicine pre-clinical courses have typically included a mix of two or more instructional formats, with frequent framing of common problems using clinical scenarios, in small group settings led by clinicians, typically preceded by an anatomy course studying the spine and extremities [27, 28], or have included musculoskeletal anatomy instruction reinforced by review of common and pertinent clinical correlations [18, 20, 29–31]. This latter description best matches our curriculum.

### The medical school’s history

Until recently, ours was the only medical school in a five state area. First year students from each of the five states took their first year basic science courses within their home state, coming to the main campus for their second year organ-system based courses. Each medical school class between 2010 and 2013 consisted of about 250 students, an increase of 70 students from 2003, counting students at all sites. Third-year clerkships were primarily conducted at the main campus, though electives were offered in each state. The introductory course in musculoskeletal medicine was originally a second-year course, residing in the organ-system curriculum, but because of staffing challenges at the outlying sites, pressure came to bear to move the course to the first year of the medical school curriculum, which occurred in the 2009-2010 academic year. USMLE Board exams were taken at the end of the second year (Step 1) and after completion of required clerkships - during the fourth year (Step 2).

An assessment of medical student knowledge and competence in musculoskeletal medicine was conducted in the early 2000’s at our institution [9]. That study revealed a generally unsatisfactory foundation in musculoskeletal medicine for the majority of our students, with the exception of those who went on to participate in clerkships in their clinical years with emphases in musculoskeletal medicine. In response, a working group was formed in 2006 to evaluate our current state and make recommendation to the Dean of Curriculum, following an evaluation and curriculum-planning model as described by Bordage [32]. Being constrained in our number of course meeting hours, and without the benefit of a required clerkship or clinical experience in musculoskeletal medicine, we sought to enrich our students’ pre-clinical experiences via institution of a number of large and small changes to our curriculum:

1. Small group meetings focused on the musculoskeletal living anatomy and common pathologies were modified via institution of a Team-Based Learning (TBL) environment for musculoskeletal clinical problem solving [33, 34], designed to take advantage of clinical instructors in small group settings with case-based scenarios. The format included the addition of a 6-8 question pre-class on-line quiz relating to clinical correlations, to be completed in class as a graded 4-student exercise, followed by larger group (12-16 students) debate and discussion. TBL became the major teaching tool used in many courses through the first two years of the medical school curriculum beginning in approximately 2008.
2. Physical exam videos of the spine and extremities were prepared by clinical instructors using professional videographers, to assist students in better learning examination of the musculoskeletal system, available at all sites through the course web-site.
3. Objective Structured Clinical Examinations (OSCEs) were introduced, more than 50% of which included a case with a musculoskeletal focus [24], given twice to second-year students and once to fourth year students.
4. A short 2-3 day required rheumatology course in the second year was instituted that included both classroom and clinical experiences.

Soon thereafter, the administration countered with the additional plan of moving the required pre-clinical course in musculoskeletal medicine from the second pre-clinical year to the first, to shift all anatomy teaching into the first year. The hope was that early exposure would lead to a foundation that could grow with future musculoskeletal experiences in pre-clinical electives and clinical clerkships.

Though all students participated in a limited clinical preceptorship during the initial two pre-clinical years during both the old and new curricula, in clinics where care of patients with musculoskeletal problems may have occurred, this exposure was neither routine nor predictable. Classroom instruction in examination of the shoulder and knee routinely occurred late in the second-year and was not routinely coordinated with the musculoskeletal system class.

With the move of the required musculoskeletal medicine course from the second to the first year, neither were the total number of contact hours changed (39 h), nor was the breakdown of hours by large-group, small group discussion, or dissection changed. The faculty remained essentially constant for approximately 50% of students. The other 50% of students obtained their musculoskeletal introductory course instruction from a cadre of basic science and clinical instructors utilizing similar educational materials at the five separate sites where our first year medical school classes are taught. The curriculum, learning objectives, and examinations remained the same across all sites. The School of Medicine is highly regarded for the training it provides students and residents in primary care, with Primary Care ranked highly for over 20 years by US News and World Report [35].

In the time since this follow-up study was conducted, the School of Medicine has radically altered the curriculum of the first two years, condensing it into an 18-month Foundations of Science training phase, combining subjects into theme-based organizations known as “blocks”, adding numerous case scenarios to each block. The timing and duration of exposure to topics in musculoskeletal medicine in this newest curriculum currently remains in flux.

We examined the results of curricular changes instituted between 2005 and 2010 on student musculoskeletal knowledge and subject proficiency, with the hope and expectation that these changes would result in an improvement in student understanding in musculoskeletal medicine. We posed the following questions:

- ○ Did the move to teaching musculoskeletal medicine to the first year of the medical school curriculum, in combination with focusing the small group experience and offering instruction and testing in the musculoskeletal examination, result in increased student knowledge and proficiency, and improve the retention of musculoskeletal knowledge into the clinical years?
- ○ Should further efforts to improve musculoskeletal medicine education focus on ensuring clinical experiences in musculoskeletal medicine?

## Methods

The academic performance of medical students from one institution was established by comparing their United States Medical Licensing Examination (USMLE) Step 1 and Step 2 overall and sub-test means with national averages for the periods 2001-2003 to 2010-2013. In addition, all students enrolled in the School of Medicine in spring quarter of 2013 were invited to participate in a musculoskeletal knowledge assessment via e-mail to all members of each medical school class. A reminder e-mail was sent to all approximately one week after the initial invitation. No incentive was offered to complete the survey. An online version of the 24-question short-answer musculoskeletal medicine survey by Freedman and Bernstein [36] was given to those electing to participate, the study having been approved by our institutional review board. The survey has been validated by 100 orthopedic program directors [36] and 240 internal medicine program directors [37] across the country. All students in the recent survey received musculoskeletal training under our new curriculum, with the introductory course occurring in the first year of medical school, followed by a focused second year rheumatology unit. Proficiency was set at 70% or greater of questions answered correctly, as previously defined [37]. The results of this survey were compared to data from the same survey obtained from medical students at our institution while the musculoskeletal course was taught during the second year of medical school over the 2001-2002 and 2002-2003 academic years [9]. Comparisons were made using a student’s two-tailed unpaired t-test for significance. The cohorts were compared by years elapsed since taking the introductory musculoskeletal medicine course, on the assumption that the greatest impact on student knowledge and proficiency in musculoskeletal topics would be related to timing of this course, as opposed to the other curricular changes.

Student preparation and readiness for the introductory unit in musculoskeletal medicine was assessed via a mean composite Medical College Admission Test (MCAT) score for each entering class. These scores were then compared between the two groups using the student’s two-tailed unpaired t-test for significance.

## Results

Between 2010 and 2013, our medical students tended to score at or near the national average on the Steps 1 and 2 of the USMLE: Step 1 overall means equal to the national average, with subtest means ranging from 0.4 standard deviations above to 0.3 standard deviations below the national averages; Step 2 overall means equal to the national average, with subtest means ranging from 0.5 standard deviations above to 0.4 standard deviations below the national averages. Results on the Musculoskeletal, Skin, and Connective Tissue Diseases subtest revealed scores equal to the national average, ranging from 0.2 standard deviations above to 0.2 standard deviations below the national average. These recent scores were perhaps slightly lower than scores ten years earlier (2001-2003), when our mean scores were greater than the national averages (at least two points or 0.1 standard deviations) on five of six Step 1 and Step 2 USMLE sub-tests, including the Musculoskeletal, Skin, and Connective Tissue Diseases subtest of the Step 2 USMLE, where scores were above the national average by 0.1 **–** 0.3 standard deviations.

**Fig. 1:**
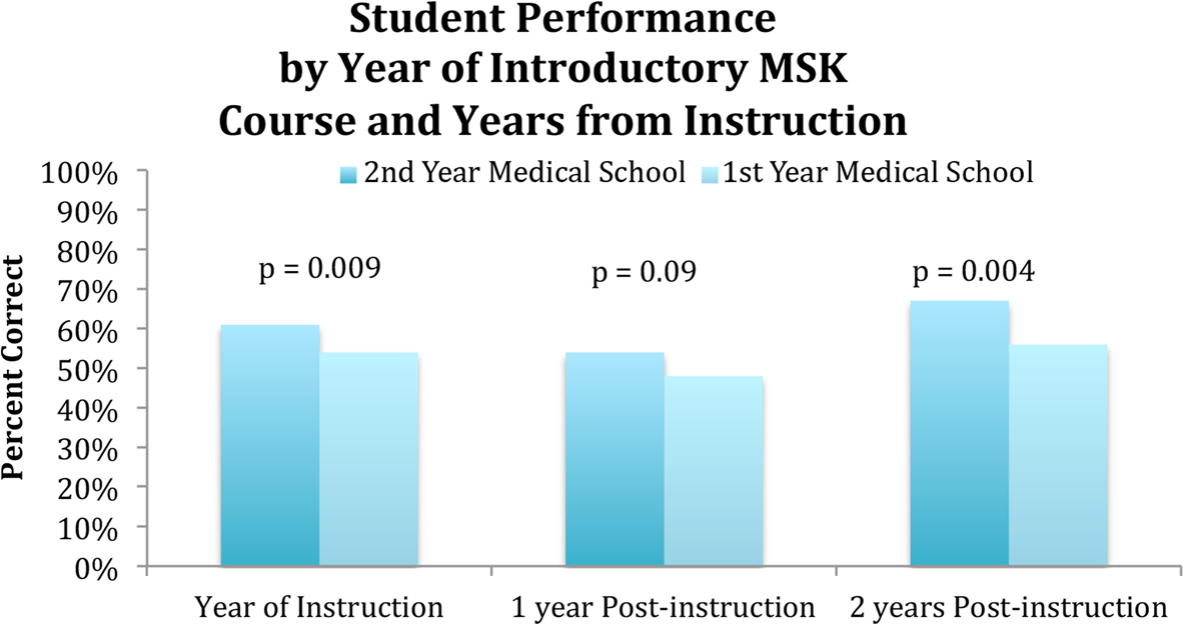
Comparison of student performance by curriculum based on time elapsed since instruction

**Fig. 2:**
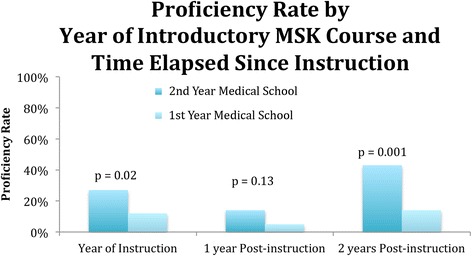
Comparison of student proficiency by curriculum based on time elapsed since instruction

The survey of student musculoskeletal knowledge conducted on-line through a web-based questionnaire had a response rate of 11% (112/986). When looking at the combined student scores, there was a statistically significant higher overall performance for students first exposed to musculoskeletal medicine during the second year of medical school compared to the those students first exposed to musculoskeletal medicine during the first year of medical school (61% correct combined overall average scores for students exposed to musculoskeletal medicine during their second years vs. 54% correct for students exposed to musculoskeletal medicine during their first years, *p* < 0.001). Only 12% of students overall were seen to achieve proficiency in musculoskeletal medicine knowledge when exposed during their first year as opposed to 27% achieving proficiency when exposed during their second years (*p* = 0.01). Students exposed to musculoskeletal medicine during their first year of medical school who were more than one year out from that course achieved an average 48% correct with 5% achieving proficiency, compared to 54% correct with 14% achieving proficiency for students at a similar stage of training first exposed to musculoskeletal medicine during their second year of medical school (*p* = 0.09 and 0.13 respectively, Figs. 1 and 2).

Students exposed to musculoskeletal medicine during their first year who were more than two years out from that course, achieved an average 56% overall correct, with 14% of students achieving proficiency, compared to 67% correct with 43% achieving proficiency for students at a similar stage of training first exposed to musculoskeletal medicine during their second year (*p* = 0.004 and 0.001 respectively, Figs. 1 and 2).

Students first exposed to musculoskeletal medicine during their first year who had taken an elective clerkship with a musculoskeletal focus during their third or fourth years scored significantly higher than students without these clinical experiences (63% vs. 53%, *p* = 0.047), similar to previous results (77% vs. 65%, *p* < 0.006) [9] reinforcing the notion that the most effective learning of musculoskeletal medicine appears to occur in the clinic.

**Fig. 3:**
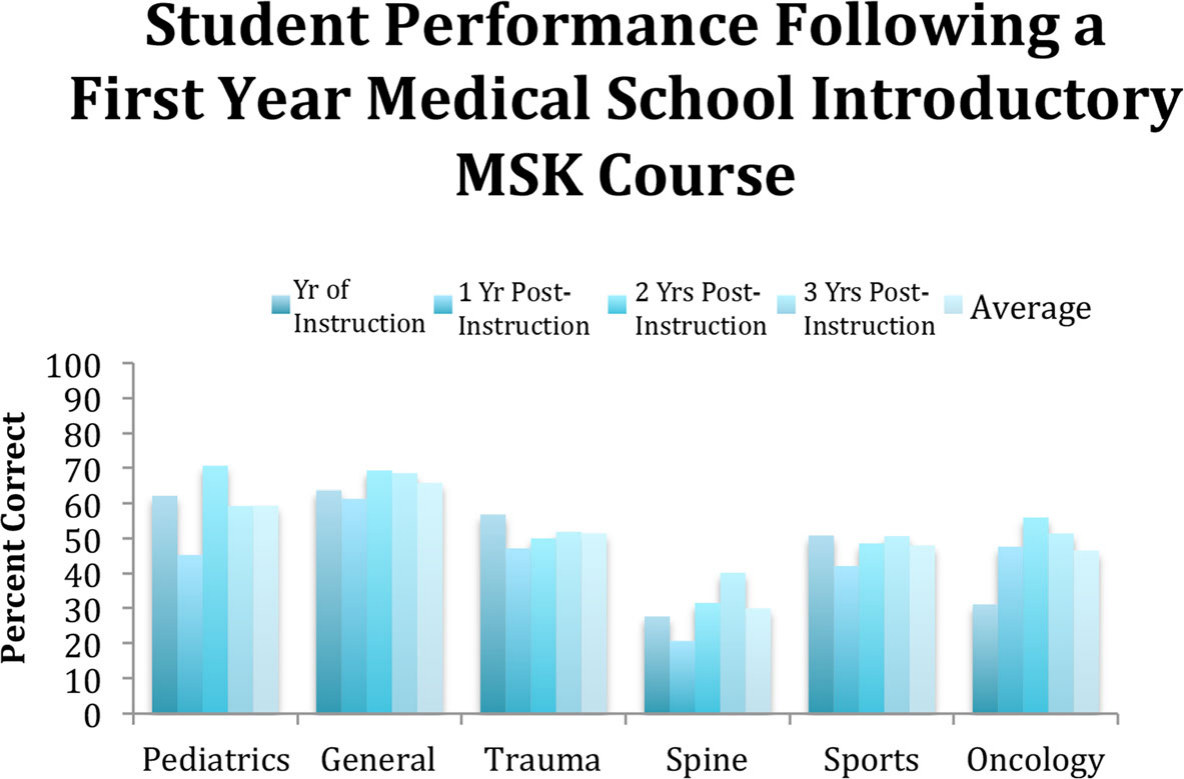
Comparison of student performance by topic based on time elapsed under the new curriculum

The addition of a two-day rheumatology-teaching event during the second year of classroom instruction was followed by a significant improvement in knowledge of arthritis. Performance on the survey’s arthritis-based question improved from 72% of students in all grades answering correctly to 91% answering correctly (*p* < 0.001). Three of the 24 questions were spine-related, including questions about back pain and disc disease. Students in both cohorts performed most poorly on spine-related questions (30% correct for spine questions vs. 54% average correct for all subject areas, Fig. 3).

The preparation of incoming medical students as assessed by their mean sub-test and aggregate MCAT scores revealed no significant differences (*p* = 0.9) between groups.

## Discussion

Numerous reports describe insufficient knowledge of important topics in musculoskeletal medicine by medical students. Though moving the introductory musculoskeletal course to an earlier year in the curriculum was not intentionally done to improve student knowledge and understanding in musculoskeletal medicine, the hope was that it would provide students with more opportunities to link concepts in musculoskeletal medicine to new learning in other basic science and organ-system courses, as well as in preclinical experiences, to reinforce and broaden understandings in ways that would be of benefit in future clinical experiences. However, with the major exposure to musculoskeletal medicine occurring in the first year of medical school, there was no evidence of improvement in student scores on the related USMLE subtest or Freedman and Bernstein’s web based questionnaire of musculoskeletal knowledge. Perhaps this was due to first year students not yet having the clinical framework needed to put pathology into context, thereby limiting mastery of the subject. We also found that earlier exposure to musculoskeletal medicine did not improve understanding in spine, which remained the weakest subject area despite the curricular change. Given the frequency with which physicians of all specialties encounter patients presenting with back pain, this suggests that greater emphasis on spine-related topics and exposure to patients with spine problems should occur during medical student training.

With the curricular changes, retention of musculoskeletal knowledge did not appear to be maintained into the clinical years. There is no evidence from this limited survey that early exposure improved student knowledge and proficiency when compared to similar assessment of students having taken the introductory musculoskeletal medicine course in the second year of medical school, though addition of a dedicated second-year short course in rheumatology did improve results on a rheumatology-based question. Many clinical faculty members informally surveyed at our institution expressed disappointment with regards to recent student readiness for interviewing, examining and diagnosing the most basic and straightforward musculoskeletal problems. Faculty comments along the lines of "It sounds like a good introductory course, but it just didn't translate into sustained knowledge" and "They never remember musculoskeletal facts and concepts by the time they get to the third or fourth year..." were typical.

The institution of numerous electronic resources, including a course web-site available to all sites, with links to video resources on physical exams, video copies of lectures, and links to additional educational resources including musculoskeletal cases with carefully worked-through differential diagnoses were not enough to improve the efficacy of the course in the face of its move to the first year.

As previously noted [9], taking specific clinical rotations with a musculoskeletal focus significantly improved student knowledge, suggesting that those who had a greater exposure to the topic in clinical settings came away with a better foundation in musculoskeletal medicine, a finding corroborated by others as well [8, 20, 36, 38–40].

### Limitations

The USMLE Musculoskeletal, Skin, and Connective Tissue Diseases subtest score includes an evaluation of understanding of skin and subcutaneous tissues as well as the musculoskeletal system, hence consideration of scores from this test as a surrogate for knowledge and understanding of facts and concepts in musculoskeletal medicine may be an oversimplification. However, as our mean scores dropped somewhat on this subtest when comparing 2001-2003 results to those of 2010-2013, one may conclude that student understanding in musculoskeletal medicine with the new curriculum is not measurably better than that student understanding with the old. This is supported by the results on the web-based survey.

Our response rate of 11% for the web-based survey is lower than most surveys in the literature, slightly lower than the 15-30% response rate described for internet or web-based surveys [41, 42] and lower than our previous survey (33%) [9], reflecting a limited sample of the student population. Errors in conducting the survey likely included sending the survey out towards the end of a grading period, with the reminder e-mail being sent around the time of the start of the next major instructional period. No incentive was offered, which may have also discouraged participation. Despite the poor response rate, one would expect that in a medical school program with criterion-referenced grading, where ideally all students would meet an accepted level of mastery of topics, knowledge scores would be above 70% and proficiency rates closer to 100% than these surveys revealed. The use of our university’s on-line system for querying students may have discouraged completion of survey by many, as the passive format of the survey made immediately apparent the large number of short-answer questions to be completed prior to submission, without offering the option of partial completion and/or delayed submission. In the case of poor response on web-based surveys, it has been argued that those responding may have a particular interest in the subject or object of the inquiry [41], potential skewing the respondent population in this survey *towards* those with an interest in musculoskeletal medicine. If that were the case here, one might argue that those responding to the survey were those with greater confidence in their musculoskeletal knowledge, and perhaps those more likely to score highly on exams such as the Freedman and Bernstein assessment.

As first year medical students at our medical school receive basic science instruction at several sites, gathering at a common site for the second year of training, multiple instructors lead the first year musculoskeletal medicine course at these various sites. Though overall student achievement in the musculoskeletal course has not varied appreciably from site to site over the past four years, as assessed by common exam results and USMLE scores, the confidential nature of our surveys did not allow us to obtain breakdown of scores by instructional site. Our data also does not allow us to assess the varying extent of exposure to musculoskeletal clinical experiences by our students during third and fourth year clerkships, except where students noted enrollment in a musculoskeletal-focused clinical elective.

There appears to be a correlation with the amount of time spent in pre-clinical musculoskeletal education with musculoskeletal knowledge. As programs have retooled curriculum that, among other changes, increased the amount of time spent studying musculoskeletal medicine, higher exam scores resulted [13, 18, 43]. This trend has not been entirely reproducible however, as the addition of an upper extremity course at one institution increased student confidence without actually increasing national exam scores [44]. We would argue that time allotted may not be as important as the timing of course delivery, as our study has revealed that earlier did not lead to better understanding nor a more utilitarian foundation for our students. Seeing how musculoskeletal concepts relate to new learning in other areas, making effective links across the curriculum to build overall understanding may require a certain background that our student did not quite have at the point of their first year course.

Integration of musculoskeletal-related topics is one way to increase mastery. A new integrated curriculum that involved more time in gross anatomy laboratories and more emphasis on orthopedic pathophysiology resulted in higher levels of clinical confidence and cognitive mastery amongst students [15]. Integrating orthopedics, rheumatology, and physical medicine and rehabilitation has been shown to be effective in increasing knowledge and retention of physical examination skills [20]. Interactive small groups are also popular amongst students in learning musculoskeletal medicine [18, 21, 45]. Such a setting may help students by breaking tasks down so that physical examination may be learned first, with exploration of clinical correlates to follow [46]. Despite student satisfaction with small groups, it is not always the case that they lead to better knowledge and understanding [22]. Computer-based instruction is also being explored, with some evidence of higher student satisfaction and skills gained [47, 48], though students prefer computer based learning to be combined with bedside teaching [49].

There are many challenges in musculoskeletal education. Student knowledge may continue to be evaluated using the Freedman and Bernstein survey, though some argue that it is more a test of orthopedic knowledge than knowledge of topics in musculoskeletal medicine. A new physical examination decision-making test has recently been developed that educators can utilize that may more effectively assess student knowledge in musculoskeletal medicine [29]. Further tests that are developed should ideally account for multiple competencies, including physical exam skills in addition to assessments of knowledge and diagnostic acumen.

## Conclusions

Given the omnipresent nature of musculoskeletal complaints across medical specialties, a greater emphasis on student preparation in topics in musculoskeletal medicine, with an increased emphasis on spine, appear to be merited. Our findings suggest that in the absence of an early organized clinical exposure to patients with musculoskeletal problems, earlier is not necessarily better when it comes to instruction in musculoskeletal medicine. A more comprehensive reform including routine early exposure to patients with musculoskeletal problems continued into the clinical years may be required to achieve optimum levels of student proficiency.

## Abbreviations

IRB: Institutional Review Board
MCAT: Medical College Admission Test
OSCEs: Objective Structured Clinical Examinations
TBL: Team Based Learning
USMLE: United States Medical Licensing Examination
